# Supplementary material for: The interactome of intact mitochondria by cross-linking mass spectrometry provides evidence for coexisting respiratory supercomplexes
Source: Mol Cell Proteomics. 2017 Dec 8;17(2):216–32. doi: 10.1074/mcp.RA117.000470 (PMC5795388; doi:10.1074/mcp.RA117.000470)
Supplement: Supplemental Data [file supp_RA117.000470_133922_0_supp_23978_fzffwf.zip › spectra_annotation/mito_DR_spectra_annotation/263-1-6-1-15-1.pdf]

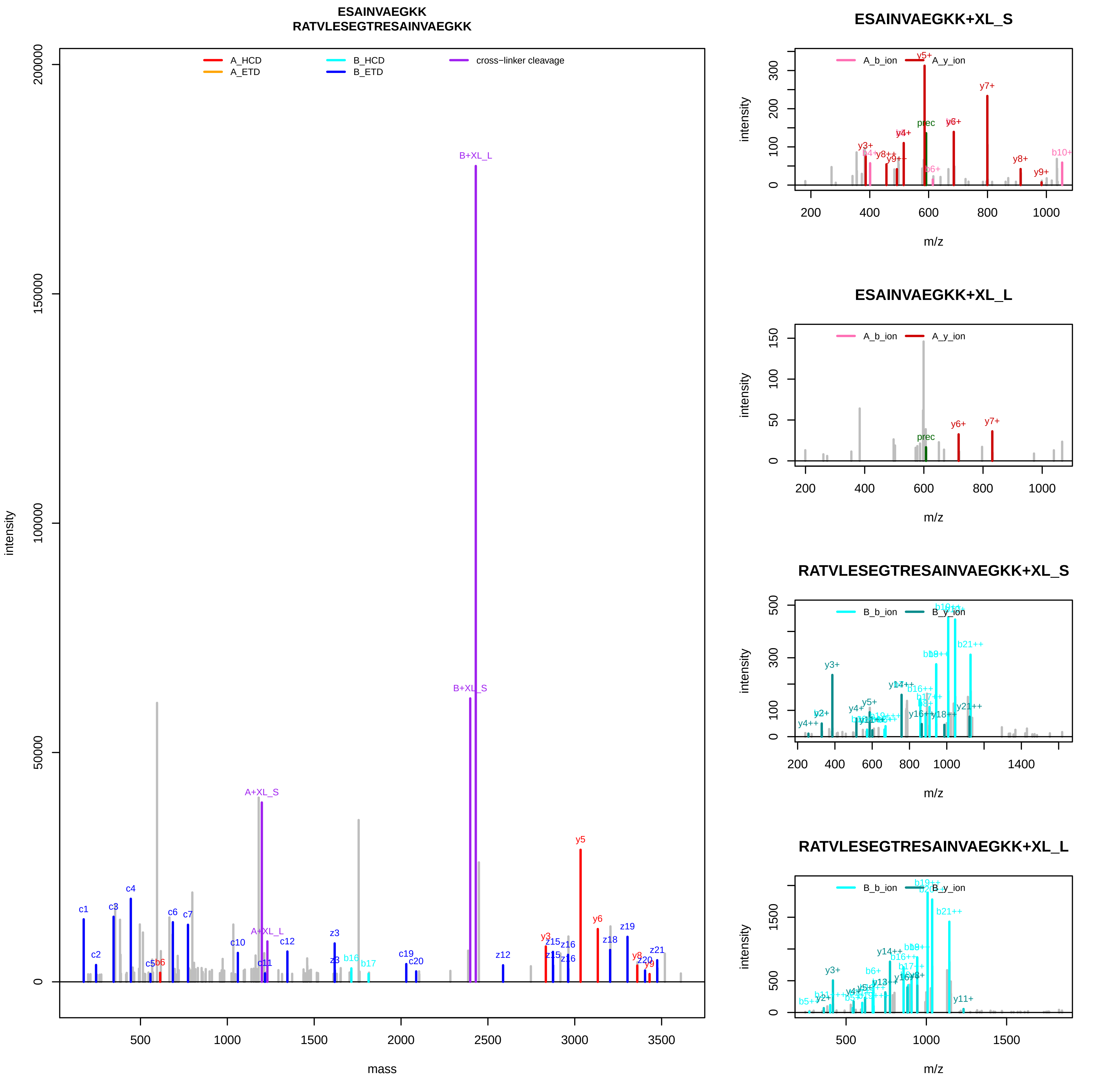

ESAINVAEGKK
RATVLESEGTRESAINVAEGKK
A_HCD
A_ETD
B_HCD
B_ETD
cross−linker cleavage
B+XL_L
B+XL_S
A+XL_S
A+XL_L
c1
c2
c3
c4
c5
b6
c6
c7
c10
c11
c12
z3
b16
b17
c19
c20
z12
y3
z15
z16
y5
y6
z18
z19
y8
z20
y9
z21
intensity
mass
0
50000
100000
150000
200000
500
1000
1500
2000
2500
3000
3500
ESAINVAEGKK+XL_S
A_b_ion
A_y_ion
y5+
y7+
prec
y6+
y3+
y4+
y8++
y9++
b4+
b6+
y8+
y9+
b10+
intensity
m/z
0
100
200
300
200
400
600
800
1000
ESAINVAEGKK+XL_L
A_b_ion
A_y_ion
prec
y6+
y7+
intensity
m/z
0
50
100
150
200
400
600
800
1000
RATVLESEGTRESAINVAEGKK+XL_S
B_b_ion
B_y_ion
y3+
y2+
y4++
y4+
y5+
y14++
b16++
b17++
b18++
y16++
y18++
b21++
y21++
intensity
m/z
0
100
300
500
200
400
600
800
1000
1400
RATVLESEGTRESAINVAEGKK+XL_L
B_b_ion
B_y_ion
b19++
b21++
y14++
b16++
b17++
b6+
y3+
y13++
y16++
y4+
y5+
y2+
b5+
y11+
intensity
m/z
0
500
1500
500
1000
1500
